# Capturing crisis dynamics: a novel personalized approach using multilevel hidden Markov modeling

**DOI:** 10.3389/fpsyt.2024.1501911

**Published:** 2025-01-14

**Authors:** Emmeke Aarts, Barbara Montagne, Thomas J. van der Meer, Muriel A. Hagenaars

**Affiliations:** ^1^ Department of Methodology and Statistics, Utrecht University, Utrecht, Netherlands; ^2^ Center of Psychotherapy, GGz Centraal, Ermelo, Netherlands; ^3^ Department of Clinical Psychology, Utrecht University, Utrecht, Netherlands

**Keywords:** crisis prevention, personality disorders, Experience Sampling Method, hidden Markov model, mobile health (mHealth)

## Abstract

**Background:**

Prevention of (suicidal) crisis starts with appreciating its dynamics. However, crisis is a complex multidimensional phenomenon and how it evolves over time is still poorly understood. This study aims to clarify crisis dynamics by clustering fluctuations in the interplay of cognitive, affective, and behavioral (CAB) crisis factors within persons over time into latent states.

**Methods:**

To allow for fine grained information on CAB factors over a prolonged period of time, ecological momentary assessment data comprised of self-report questionnaires (3 × daily) on five CAB symptoms (self-control, negative affect, contact avoidance, contact desire and suicidal ideation) was collected in twenty-six patients (60 measurements per patient). Empirically-derived crisis states and personalized state dynamics were isolated utilizing multilevel hidden Markov models.

**Results:**

In this proof-of-concept study, four distinct and ascending CAB-based crisis states were derived. At the sample level, remaining within the current CAB crisis state from one five-hour interval to the next was most likely, with staying likeliness decreasing with ascending states. When residing in CAB crisis state 2 or higher, it was least likely to transition back to CAB crisis state 1. However, large patient heterogeneity was observed both in the tendency to remain within a certain CAB crisis state and transitioning between crisis states.

**Conclusion:**

The uncovered crisis states using multilevel HMM quantify and visualize the pattern of crisis trajectories at the patient individual level. The observed differences between patients underlines the need for future innovation in personalized crisis prevention, and statistical models that facilitate such a personalized approach.

## 1 Introduction

Admissions (voluntary and involuntary) to acute (suicidal) crisis services are a common phenomenon in the treatment of severe psychological disorders. Although they may be necessary in some cases, they have been shown to have adverse (long-term) effects, and their effects vary widely among patients (1, 2). Prevention of (suicidal) crises should therefore be a crucial element in the treatment of crisis-sensitive psychological disorders, such as personality disorders (PDs; 3). Although (suicidal) crisis has been described most often in borderline and antisocial PDs, other PDs have also been associated with increased risk (4, 5). This makes sense given that the three main models of (suicidal) crisis include impaired social connectedness and belonging as an important risk factor (6–8), which play a role in many PDs. Detailed information about crisis emergence is thus key to effective prevention in PD. However, how crises evolve over time is still poorly understood (6). Little is known about fine-grained variability over time outside the context of treatment. How do patients move from a low symptom state to a crisis state? It has been proposed that crisis-sensitive patients are unstable, but does this literally mean that they move quickly into crisis, or do we simply not yet understand this “transition”? The field also lacks knowledge about how patients “transition” from crisis to more stable states.

Crisis is a complex interplay of several factors that interact over time (6). In addition to suicidal ideation, the multidimensionality of (suicidal) crises seems to be reflected in symptoms on different levels: Cognition (e.g., loss of [self]control), Affect (e.g., negative mood), and Behavior (e.g., social withdrawal; CAB 9–11). The interplay between crisis-related symptoms are known to be heterogeneous in patients with PD. That is, different individuals may have different combinations of suicidal ideation and (CAB) symptoms. Using latent class or profile analysis, several at-risk subtypes have indeed been identified (see e.g., 12, 13). These findings illustrate the heterogeneity between individuals, but do not provide insight into variation over time.

In addition to differences in the expression of (suicidal) crisis between individuals, the expression of (suicidal) crisis also varies over time within the same individual. For example, the Joint Crisis Plan (JCP; detailing a psychiatric advance directive that includes the formulation of predictive signs and management strategies for crises 14) assumes the existence of ascending crisis states that alternate over time within patients. The idea is that patients move through successive stages, becoming progressively worse in each stage. To model individual differences in symptom development, researchers have used growth mixture models and latent class trajectory models (e.g., 15). For example, after aversive events, some people exhibit stable low or stable high symptom profiles, while others experience a delayed onset of symptoms, or a decline from initially high symptom levels (16). While these models do provide some insight into the development of crises over time and differences between individuals herein, the information obtained on the dynamics of suicidal crisis over time is still limited. That is, using growth mixture models, the process under study is characterized by a single set of parameters for each subgroup, e.g., a baseline level of crisis, and a linear progression of crisis over time (17). If an individual is classified as being in the subgroup of increasing suicidal crisis expression, these models cannot account for how an individual may change to a decreasing trend, or how individuals may alternate between periods of high and low crisis expression.

In order to gain detailed insight into individual crisis dynamics over time, intensive longitudinal measurements of possible crisogenic factors can be obtained using the Experience Sampling Method (ESM; 18). In ESM, participants frequently report on symptoms, (social) behaviors, or emotions in their daily lives. ESM uniquely allows the study of psychological processes over time at the within-person level (and between-person differences therein; 19), while minimizing recall bias and increasing ecological validity (20). Kleiman et al. (21) used digital smartphone monitoring to examine suicidal thinking and found five distinct subtypes that differed in severity (i.e., mean suicidal thinking) and how much individuals varied in their suicidal thinking over time. Those with more severe but also less variable suicidal thinking were most likely to actually attempt suicide. To increase insight into crisis dynamics and symptom interplay over time, a next step would be to account not only for differences in severity between individuals, but also for differences in suicidal thinking over time within individuals, and to include other crisis-relevant (i.e., CAB) symptoms.

A natural model for identifying within-person differences over time in the expression of suicidal crisis is the hidden Markov model (HMM; 22, 23). The HMM is a longitudinal latent mixture modeling approach that models the dynamics of an individual as switching between a number of hidden (i.e., latent) discrete states. With the HMM, the evolution of cognitive, affective, and behavioral factors over time is partitioned into empirically derived CAB crisis states. Thus, the HMM allows for multidimensional crisis states that vary over time within patients. By inferring the probability of transitioning between hidden states over time, the HMM additionally uncovers the dynamics in CAB crisis states. By adopting a multilevel framework (24), patient heterogeneity in CAB crisis state composition and dynamics is accommodated, allowing for both a model at the sample level representing the overall dynamics over time and fully personalized patient models.

The present proof-of-principle study aims to identify personalized patterns in crisis dynamics based on ESM data. Specifically, we test whether ascending crisis states can be detected based on a complex of symptoms at the cognitive, affective, and behavioral levels CAB: self-control, negative affect, contact avoidance, contact desire, and suicidal ideation). In addition, we infer crisis dynamics at both the group and individual patient levels. We expect to find at least two CAB states. Given the complexity of crisis dynamics, we also expect large individual differences in the transition between the identified states. Data were collected on twenty-six patients (60 measurements per patient).

## 2 Materials and methods

### 2.1 Participants

Thirty crisis-sensitive patients (80% female) who received outpatient treatment in the Netherlands for varying personality disorders (PD) participated. Four patients dropped out due to motivational problems during data collection. The final sample included 26 patients between the ages of 20 and 59. Demographic characteristics are presented **Supplementary Table S1** in the Supplementary Material. We did not collect data on the ethnic and cultural backgrounds of our participants and thus cannot be sure that our sample is representative of demographic groups in The Netherlands. Patients met the full (n = 23) or subthreshold (n = 3) DSM-5 criteria for at least one PD. Of the 26 patients diagnosed with a PD, 9 had borderline PD, 10 unspecified PD, 5 avoidant PD, 1 dependent PD and 1 obsessive-compulsive PD. Note that although comorbidity with another PD classification was low (n=3), comorbidity with non-PD classifications was high, see **Supplementary Table S1** for an overview. Participants who did not own a smartphone, had an IQ below 80, and/or were diagnosed with a psychotic or bipolar disorder were excluded from the study. The study was approved by the local Medical-Ethical Testing Committee of Maastricht UMC (non-WMO declaration 2018-0649 METC azM/UM) and was conducted in accordance with relevant laws and institutional guidelines.

### 2.2 Procedure

All participants were informed about the research at the start of their outpatient treatment. They subsequently received the information letter. Patients who agreed to participate signed the informed consent. After signing informed consent, each participant created a (or updated or used an already existing) JCP (25) with the researchers’ assistance, to be used as ESM-items. Each JCP was phrased in personalized language to increase commitment and to better represent the experiences of each individual participant. A complete overview of the JCP including personalizing options is provided in the online supplemental material. Participants completed the ESM-items online on their smartphones three times per day, after receiving a text with a link. The interval between these three measurements was the same for each participant: 5 hours between the first and second measurement and 5.5 hours between the second and third measurement. Timing of the first-day measurement was personalized, mean starting time = 10:25 (SD: 0.71; range: 8:00 - 11:30). Participants had to complete each measurement within one hour (and received one reminder text after 30 minutes) before the measurement was registered as missing.

Participants were finished after completing 60 measurements (independent of the number of missing measurements), with a mean duration of 26.24 days (SD = 4.80, range = 20.33-37.67 days). The mean number of missing measurements was 14.23 (SD: 13.12; range: 1-53). The total number of observations in this study (excluding missing measurements) equals 26 × 60 = 1560 observations per CAB symptom. Previous research on multilevel HMMs has shown a compensation effect between the number of participants and the number of assessments, resulting in reliable parameter estimates when multivariate continuous outcomes are modeled as Gaussian distributions with the number of participants equal to 30 with each 50 observations (26).

### 2.3 Measures

We selected specific symptoms per CAB-level that had previously been specified as symptoms of acute (suicidal) crisis: self-control (27, 28), negative affect (e.g., negative emotions or emotional pain 10, 29), contact avoidance and desire (30, 31), and suicidal ideation. All items were scored on a scale from 1 (“not at all”) to 100 (“very much”). Momentary *self-control* reflected the cognitive domain, and was assessed by one ESM item: “At this moment I experience control”. Examples of personalizing “I experience control” were “I feel in control” or “I oversee my life”. Momentary *negative affect* reflected the affective domain, and was assessed by the average of four ESM items: “At this moment I feel angry/sad/afraid/tense”. The two types of social behavior (*contact avoidance* and *contact desire*), and *suicidal ideation* all reflected the behavioral domain. Contact desire and contact avoidance were both assessed by one ESM-item “At this moment I desire contact with others” and “Since the last measurement I avoided contact with others”, respectively. Suicidal ideation was assessed by one ESM-item (“Since the last measurement I thought about suicide”).

### 2.4 Multilevel hidden Markov model

**Figure 1:**
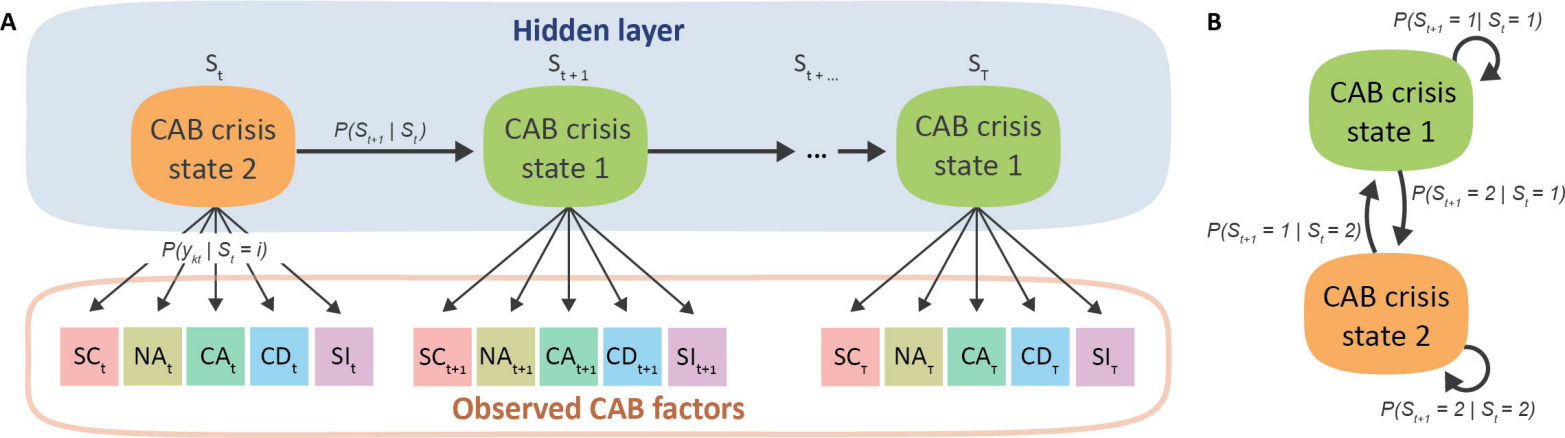
Graphical illustration of the hidden Markov model (HMM) panel **(A)** and temporal dynamics between isolated CAB crisis states panel **(B)** for an example of a two-state HMM. Here, the hidden (i.e., latent) CAB crisis states S∈ (1,2) over time t∈ (1,2,…,T) are inferred by the observed CAB symptoms self-control (SC), negative affect (NA) contact avoidance (CA) and desire (CD) and suicidal ideation (SI).

A Bayesian multilevel HMM (see e.g., 32, 33) was used to identify subsequent CAB crisis states (i.e., “hidden states”) in patients with PD. The HMM is a probabilistic model used to infer hidden states $S_{t}\in \;\left(1,\;2,\ldots ,\;m\right)$ at each time point t= 1, 2,…, T . The hidden states are defined by the probability to observe an outcome *Y_t_*, and account for the dynamics of the observations in terms of the dynamics of the hidden states. The former is based on the assumption that a given observation *Y_t_* in the sequence is generated by an underlying, latent state *S_t_*. The latter is based on the assumption that the hidden states follow a Markov process. That is, the probability of switching to the next state $\;S_{t+1}$ only depends on the current state *S_t_*. See **Figure 1** for a graphical illustration, and the online supplemental material for a more detailed model specification.

When applied to cognitive, affective, and behavioral processes, the HMM can be used to quantify information on the latent temporal dynamics into two sets of parameters: 1) the probability of transitioning between each of the latent (e.g., crisis) states $P\left(S_{t+1}=j|S_{t}=i\right)$ and 2) the probability of emitting an observation given the current latent state $P\left(y_{kt}|S_{t}\right)$ . Given that our CAB factors are continuous variables, we assume normally distributed emission probabilities. The multivariate data is accommodated by assuming that the dependent variables are conditionally independent given the sequence of hidden states. Hence, in the current study, hidden states represent crisis states inferred from the ESM CAB symptom measurements self-control, negative affect, social behavior, and suicidal ideation simultaneously.

By adopting a multilevel framework, the HMM is extended to the mixed-effects framework (24), allowing for parameter estimates at both the sample- and patient individual level. Here, observations (level 1) are assumed to be nested within patients (level 2). Within this framework, the overall crisis dynamics are reflected by a set of group-level parameters, and variability between patients is accommodated by the inclusion of continuous patient level random effects. As such, sample-level parameters were based on the pooled ESM measures, and patient individual-level parameters were subsequently sampled from the group-level distributions. Hence, while each patient is allowed to have its own parameter values, these were all obtained from one model simultaneously fitted to all data, with all patients fitted by the same number and similar composition of the CAB crisis states. As our analyses were exploratory in nature, we did not make specific hypotheses.

### 2.5 Statistical analysis

All statistical analyses were performed in the open-source software package R V4.3.3 (34). The R package mHMMbayes (35) version 1.1.0 fitted a multivariate multilevel HMM over the five CAB factors. The code to reproduce the fitted models and post-process the obtained results can be found at (36). The model was run with 10,000 iterations and a 5,000 burn-in period and weakly informative priors. Missing data was accommodated in the model and treated as Missing at Random (37), here implying that the frequency of missingness is independent of the hidden states given the observed data and model parameters.

Models with two to five CAB crisis states solutions were fitted, model selection was performed on lowest Akaike Information Criterion (AIC), model convergence, and interpretability. The ability of the model to reproduce the original ESM data was checked via Bayesian posterior predictive checks (PPCs; for more details see: 38). Goodness of fit was further evaluated by examining the distribution, homoscedasticity, and autocorrelation of pseudo-residuals for each patient on the final model by visual inspection. Convergence of all sample-level parameters was checked with the potential scale reduction factor $\hat{R}<1.20$ for two additional chains with varying starting values (39). The Viterbi algorithm (40) was used to uncover each patients’ personalized sequence of CAB crisis states.

## 3 Results

### 3.1 Characterization of CAB crisis states by self-control, negative affect, social behavior, and suicidal ideation

The four-state multilevel HMM showed the best fit indicated by the AIC (**Supplementary Table S2**), with AIC values increasing beyond the four-state model. In addition, model preference is based on state composition: the four-state model adds a well separated low crisis state not present in the three-state model (**Supplementary Table S3**). All models show good model convergence with $\hat{R}$ values well below 1.2 (**Supplementary Table S2**). Posterior predictive checks for the four-state model revealed adequate fit for group-level means and standard deviations of the CAB factors (**Supplementary Figure S2**) and pseudo-residuals generally exhibited adequate distributional properties across patients (**Supplementary Figure S3**–**S7**).

**Figure 2:**
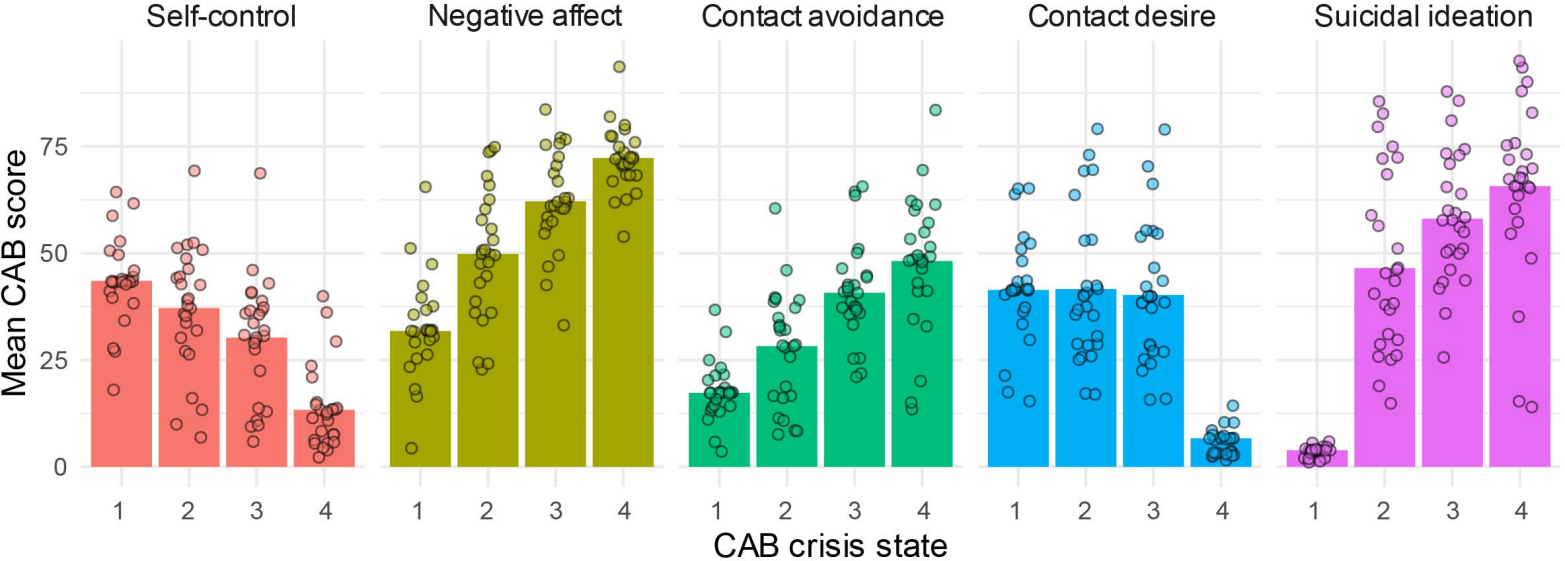
CAB crisis state composition by self-control, negative affect, contact avoidance, contact desire and suicidal ideation. Crisis state dependent means are displayed at the sample-level (bars) and patient individual level (dots).

The four uncovered crisis states show progressively worse crisis states as illustrated in **Figure 2**. That is, the crisis states are characterized by a decreasing self-control score (M = 43.53, 37.16, 30.25 and 13.30, respectively) and increasing negative affect (M = 31.80, 49.84, 62.15 and 72.31, respectively) and contact avoidance scores (M = 17.29, 28.23, 40.75 and 48.17, respectively). In addition, crisis state 1 is characterized by a distinctively low suicidal ideation score (M = 3.85) in comparison to crisis state 2 and 3 (M = 46.51 and 58.07, respectively) and crisis state 4 shows the highest suicidal ideation score (M = 65.71) albeit with a smaller difference. Moreover, crisis state 4 is characterized by a distinctively low contact desire score (M = 6.67), while the contact desire score remains equivalent over crisis state 1 to 3 (M = 41.38, 41.59, and 40.22, respectively).

Patients show a large variability in mean CAB measure scores over the states (See **Figure 2**). However, the above-described pattern in CAB crisis state characterization is persevered in almost all patients (i.e., patients generally scored lower/higher on a CAB symptom over all four states, see **Supplementary Figure S8**). This indicates that the CAB crisis states determined at the group-level can be interpreted similarly across patients, while accommodating heterogeneity between patients.

### 3.2 Dynamics in CAB crisis states

#### 3.2.1 Remaining within the current CAB state

**Figure 3:**
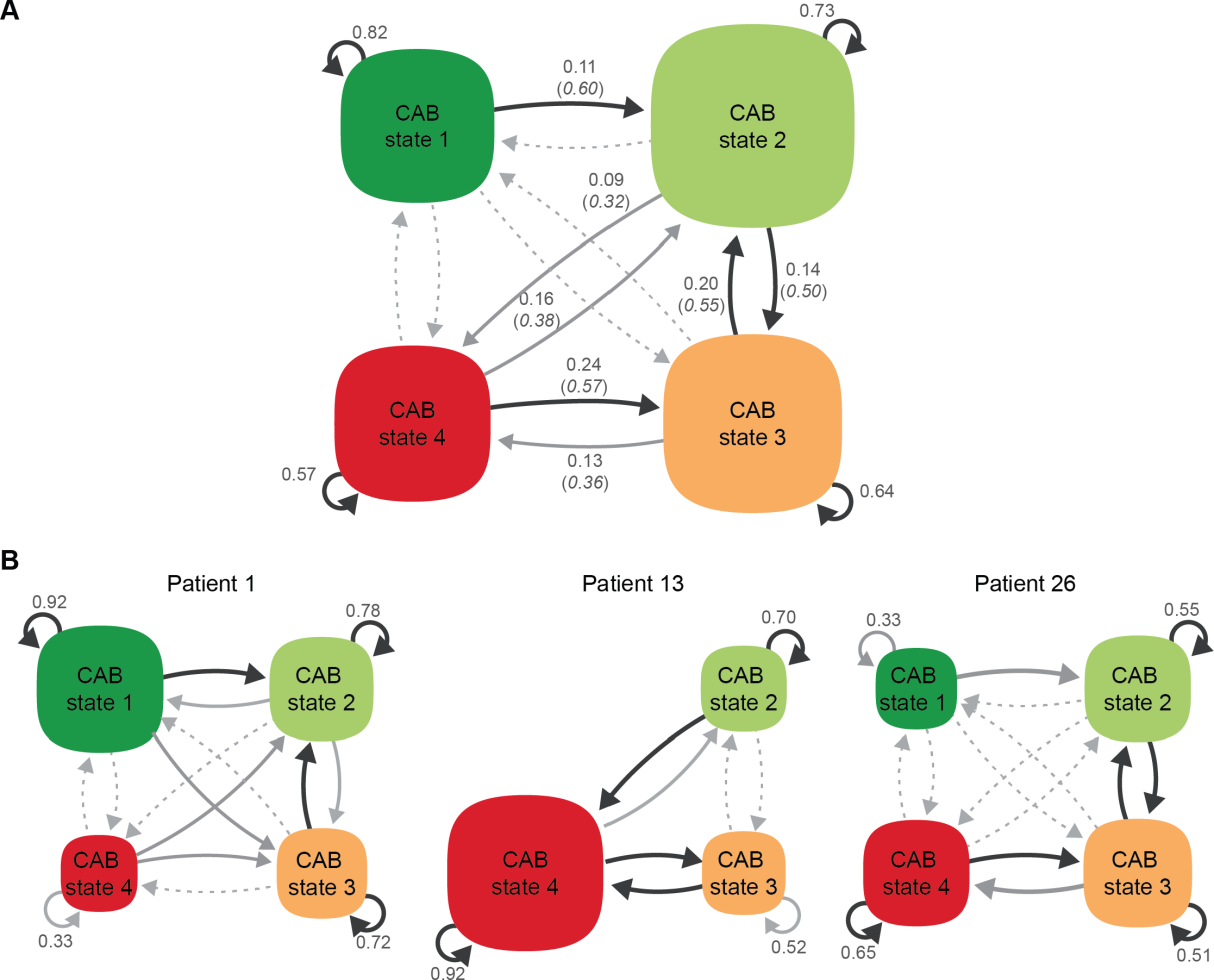
Graphic representation of the CAB crisis state dynamics at the sample-level panel **(A)** and patient-individual level panel **(B)**. CAB crisis states are depicted in squircles, with the area of the squircles proportional to the incidence of each CAB state. Arrows that loop back to the departing state represent probabilities to remain within the current state from one occasion to the next. Arrows pointing toward another state represent probabilities to switch to another CAB state, with type and color indicating the magnitude of the normalized switching probabilities (i.e., discounting probabilities to remain within the current CAB state to allow for the comparison of between state transition probabilities over transitions departing from different states). Dashed lines indicate relative probabilities < 0.3, gray lines relative probabilities on the range 0.3 − 0.5, and black lines relative probabilities > 0.5. In panel **A**, absolute probabilities are indicated next to the arrows, standardized transition probabilities are indicated in italics in between brackets. Only normalized switching probabilities > 0.3 have been labeled. In panel **B**, CAB crisis state dynamics are shown for: a patient with a relatively high incidence of and probability to remain within CAB state 1 (Patient 1), a patient with a relatively high incidence of and probability to remain within CAB state 4 (Patient 13), and a patient that switched relatively quickly between CAB states from one occasion to the next (i.e., none of the probabilities to remain within the current state was ≥ 0.7; patient 26). In patient 13, CAB state 1 is not is visited in the inferred state sequence and is omitted from the graphic representation of patient 13.

On the sample level, for each crisis state it was most likely to remain within the current CAB crisis state from one five-hour interval to the next, with staying likeliness decreasing with ascending states (state 1: 84%, state 2: 73%, state 3: 63%, state 4: 57%), see **Figure 3A**. At the patient level, we observed large heterogeneity in the probabilities of remaining within the current state, see **Figure 3B** for three examples and **Supplementary Figure S9** for all patient individual specific state dynamics parameters). Classifying probabilities to remain withing the current state as ‘high’ if the probability 0.70, we observed the following pattern. Most patients had high staying probabilities for only one (sixteen patients) or two states (three patients). Which of the four CAB states had the highest staying probability varied over patients. Nine patients had a high staying probability for CAB state 1 (probability ranging from 0.79 - 0.99; patient 1, 10, 11, 14, 15, 16, 17, 19, 24). Eight patients had the highest staying probability for CAB state 2 and/or 3 (probability ranging from 0.71 - 0.92; patient 1, 2, 4, 5, 6, 9, 15, 23). Another four patients had the highest probability to remain within state 4 (probability ranging from 0.76-0.98; patient 12, 13, 18, 21, 22). In six patients, none of the probabilities to remain within the current state reached the threshold of 0.70 (maximum probability within a patient ranging from 0.50 to 0.68); these patients switched relatively quickly between CAB states from one five-hour interval to the next (patient 3, 7, 8, 20, 25, 26).

#### 3.2.2 Switching between CAB states

When switching to another state, we observed the following at the sample level (see **Figure 3A**). From CAB state 1, it was most likely to switch to CAB state 2 instead of directly switching to CAB state 3 or 4. The dynamics between CAB state 2 and 3 showed a reciprocal relation: from CAB state 2, it was more likely to transfer to a higher CAB state, i.e., CAB state 3 or 4, instead of switching back to CAB state 1. From CAB state 3, it was most likely to switch back to CAB state 2. From CAB state 4, it was most likely to switch back to CAB state 3, followed by CAB state 2.

At the patient individual level, the sample state switching dynamics were replicated as follows. Predominantly switching from CAB state 1 to either state 2 or 3 was observed in all patient-individual dynamics except for patients 16 and 25. Switching from CAB state 2 to a higher CAB state was observed in most patient individual level dynamics; in patients 1, 3, 10, and 11, 16 it was more likely to switch from CAB state 2 to state 1 instead. Predominantly switching from CAB state 3 to state 2 was observed in fourteen out of 26 patients. In eight patients, it was more likely to switch up to CAB state 4 instead of switching to a lower CAB state (patients 7, 8, 12, 13, 16, 18, 21, and 25). In the remaining four patients, state 3 was not observed in the inferred state sequence. Predominately switching from CAB state 4 to state 3 was observed in fifteen out of 26 patients. For three patients, it was more likely to switch to CAB state 2 instead of 3 (patients 1, 3, and 22). For the remaining patients, CAB state 4 was not observed in the inferred state sequence.

#### 3.2.3 Individual CAB crisis state trajectories over time

**Figure 4:**
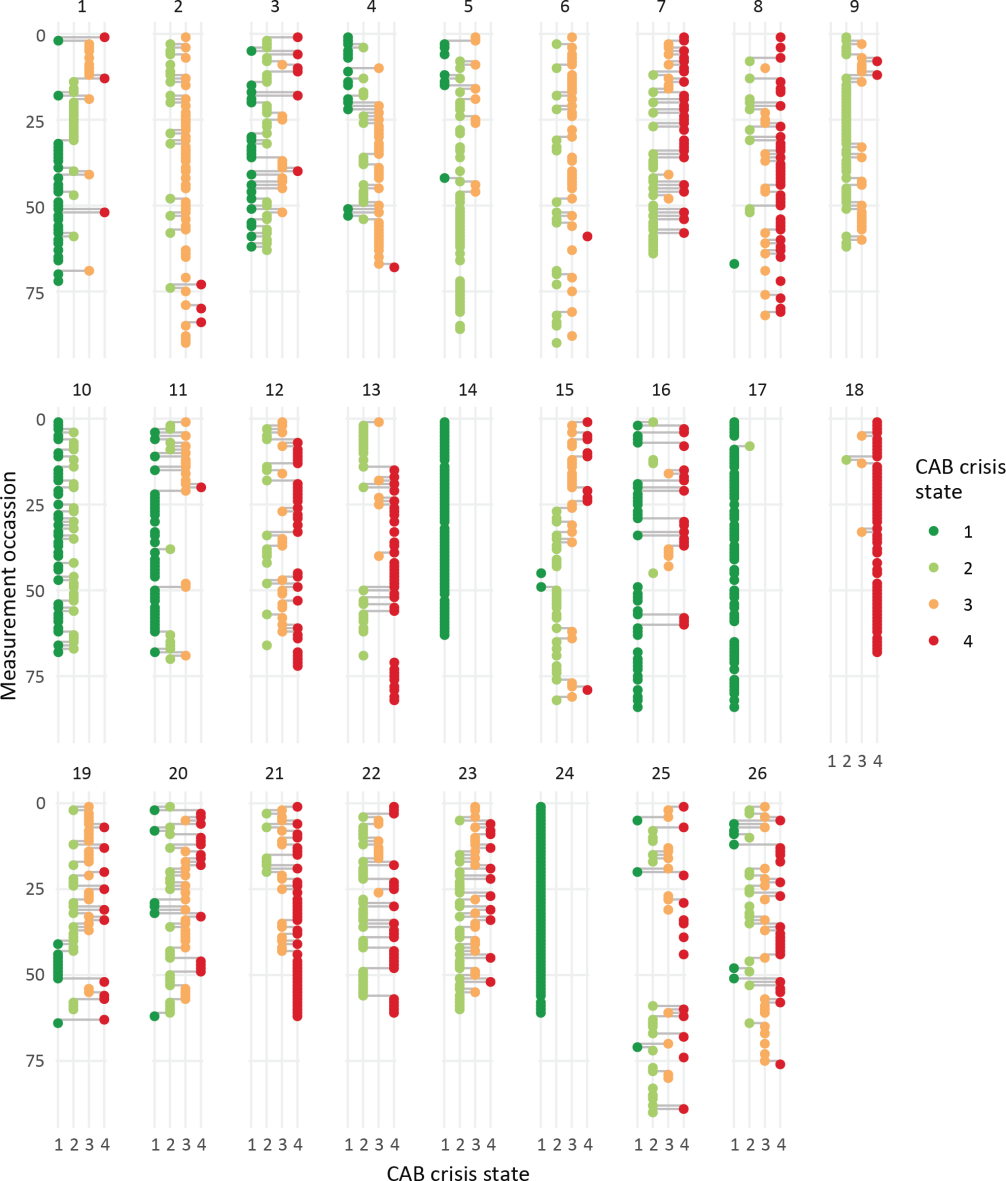
Individual CAB crisis state trajectories over time. CAB crisis states (x-axis, color coded dots) over measurement occasions (y-axis) for each of the patients (panels).

**Figure 4** visualizes the most likely CAB crisis state at each point in time for all patients. The state trajectories over time result from combining the patient individual models with the patients’ observed data. Again, high patient heterogeneity is exemplified. For example, some patients spent most of their time in crisis state 1 (patient 1, 10, 11, 14, 16, 17, and 24), which aligns with patients displaying high probabilities of remaining within CAB state 1. Yet, other patients spent most of their time in crisis state 4 (patient 7, 8, 12, 13, and 21). In addition, the distinction between patients with and without high staying probabilities is visually underlined: some patients make very little switches, e.g., patient 14 and 24 remain in state 1, and patient 5 and 9 remain predominantly in state 2, with hardly any transitions. On the other side of the spectrum, patients 20 en 26 for example make frequent switches from all of the states.

## 4 Discussion

With this explorative study, we aimed to show how complex crisis patterns over time can be identified using multilevel HMM. Applied to patients characterized by a high likeliness of suicidal crisis, we found four distinct cognitive, affective, and behavioral (CAB) crisis states as well as specific dynamics herein on a group level and on a patient personalized level. If replicated in larger samples, the identification of these ascending crisis states may facilitate early detection of emerging crisis as well as the use of patient specific intervention of CAB factors.

### 4.1 CAB factors

Our findings corroborate models of (suicidal) crisis that underscore the complexity of pathways leading to crisis (6). That is, not only did the five CAB symptoms vary over the four crisis states, they also showed distinct patterns. Self-control showed a linear decrease, while negative affect and contact avoidance showed a linear increase over subsequent states. Contact desire and suicidal ideation, however, showed different patterns. In the first three crisis states participants desired social contact, but they gradually started to avoid it. Contact desire was strongly reduced in CAB crisis state 4 only. Social factors have been indicated to play an important role in crisis emergence before (3). For example, social isolation, a low sense of belonging and perceived loneliness were found to be risk factors for suicidal crises (30, 31). Our findings suggest that when crisis is nearer, patients still feel the desire to be in contact, but they avoid it increasingly. Also, our data suggest that when social desire does decrease, patients are already in the final (worst) CAB state. Our findings may underscore the importance of active involvement of significant others of PD patients when implementing (preventive) crisis interventions. Suicidal ideation levels strongly increased from crisis state 1 to 2 and only marginally in subsequent crisis states, suggesting that in our sample, crisis states in patients with personality disorder are defined by more than just suicidal ideation.

### 4.2 CAB dynamics (overall and personalized)

Our approach provides insight in crisis state dynamics. On the sample level, it was most likely to remain within the current CAB crisis state from one five-hour interval to the next, instead of transitioning to a higher or lower crisis state. Typical transitions from CAB state 1 were to state 2 and state 3, indicating that a relatively quick worsening of symptoms was as likely as gradual crisis development, although switching from state 1 to state 4 was less likely. The same was true for CAB state 2, patients were most likely to switch to state 3 rather than back to state 1. When residing in CAB crisis state 3 it was most likely to transition back to CAB crisis state 2, instead of switching up to CAB state 4. Typical transitions from CAB state 4 were to state 3 and 2, a gradual and quick descending, respectively. Together, these findings suggest that crisis can develop gradually (state 1 → state 2 → state 3), but may also emerge relatively quick (state 1 → state 3). The same is true for those in crisis: recovery may come gradual (state 3 → state 2) but also fast (state 4 → state 2), although chances of going to the most favorable state (state 1) directly are low. This is in line with the findings of Kleiman et al. (21), who found large differences between participants in the variation of suicidal thoughts over time. We showed that this was also the case for other crisis-related symptoms. In addition, we identified specific patterns for specific symptoms as well as typical transitions between crisis states.

However, large heterogeneity was observed in the CAB crisis state dynamics between patients, as reflected in both the individual-specific state transition probabilities and the visualized individual CAB crisis state trajectories over time. Sample-level CAB dynamics should therefore be interpreted with caution. Although at the sample level the probability of remaining in the current CAB state decreased across subsequent CAB crisis states, the crisis state with the highest probability of remaining in the current state varied across individuals. In addition, the stability of states over time also showed great variability. On the one hand, some patients barely transitioned or did not transition at all, and on the other hand, some patients transitioned almost continuously. Regarding switching between CAB states, switching from crisis states 2 and 3 showed particularly high variability. When exiting crisis state 2, about 80% of the individuals switched to a higher CAB crisis state, while 20% of the individuals tended to switch to crisis state 1. When exiting crisis state 3, about 64% of the individuals switched down, while the remaining 36% were more likely to switch up to CAB crisis state 4. When exiting crisis state 4, the vast majority of individuals switched to CAB crisis state 3.

### 4.3 Strengths, limitations, and future directions

The current study is innovative in several respects. First, the intensive ESM provides many observations per individual, allowing the study of the (suicidal) crisis process unfolding over time at the individual level. Second, the multilevel HMM is a state-of-the-art method that makes optimal use of intensive longitudinal data by allowing simultaneous modeling of crisis states, their temporal stability, and transition probabilities to other states at the personalized level. In contrast to machine learning methods that typically aim at optimized prediction (e.g. of suicidal behavior) at the expense of model interpretability, the multilevel HMM provides an intuitive description of the (suicidal) crisis process unfolding over time. Previous approaches to observed CAB factors applying HMM methodology used relatively infrequent symptom assessments (e.g., retrospective weekly or monthly symptom questionnaires and clinical interviews), limiting results to group-level averages and obscuring fine-grained information on the personalized dynamics over time. In addition, these studies focused on disorder specific symptomatology states instead of more general CAB-based states, such as depression (41, 42) or bipolar states (43–45). To our knowledge, only one study did apply similar statistical methodology to fine-grained, prolonged observations of affective and behavioral experiences, albeit in a sample of college students instead of a PD sample (46). Here, (multilevel) HMMs were used to derive latent psychological distress states.

Our study also has several limitations. First, although the total sample size (number of patients × number of assessments) is comparable with previous studies, the limited number of patients restricts the generalizability of our results, and replication is merited. A larger sample would also allow investigating whether observed heterogeneity in crisis dynamics can be (partially) explained by covariates such as medical condition. Second, one should be cautious with assuming a general four state model of crisis, as the individual experiences of crisis state dynamics and trajectories differ considerably. Third, the multilevel HMM implicitly assumes equally spaced measurements. However, a longer time-gap is present between the last and the first measurements of consecutive days (so overnight) in the ESM data. Not accommodating the unequally spaced measurements may have introduced noise in the estimation of the transition probabilities. Lastly, crisis is a complex and multidimensional concept. Future research must focus on validating derived latent crisis states. In addition, future research may also link the observed heterogeneity in CAB crisis state dynamics to patient level characteristics such as type of PD, and isolate (sub)groups at larger risk of switching toward worse crisis states. For example, the identified crisis states may be linked to outcomes such as treatment effects, follow-up suicide attempts or emergency consultations.

### 4.4 Conclusion

In conclusion, using finegrained ESM data we were able to identify four distinct crisis states based on CAB factors, as well as typical transitions between the states in a real-world clinical setting. The uncovered crisis states using multilevel HMM quantify and visualize the pattern of crisis trajectories, holding the promise to quantify CAB crisis dynamics on a patient individual level. The considerable variation between patients observed in both the tendency to remain within a certain CAB crisis state and transitioning between crisis states highlights the need for a personalized method. This underlines the need for future innovation in personalized crisis prevention, and statistical models that facilitate such a personalized approach.

## Data Availability

Interested researchers obtain de-identified data after signing a Data Use Agreement and obtaining IRB approval. Requests to access the datasets should be directed to Barbara Montagne, b.montagne@ggzcentraal.nl.
