# Supplementary material for: Capturing crisis dynamics: a novel personalized approach using multilevel hidden Markov modeling
Source: Front Psychiatry. 2025 Jan 14;15:1501911. doi: 10.3389/fpsyt.2024.1501911 (PMC11772436; doi:10.3389/fpsyt.2024.1501911)
Supplement: Supplementary file 1 [file DataSheet1.pdf]

Supplementary Material

1 BAYESIAN MULTILEVEL HIDDEN MARKOV MODEL SPECIFICATION

The HMM is a statistical method that is used to infer a sequence of latent or hidden states $S_t \in (1, 2, ..., M)$ for time points $t = 1, ..., T$. The hidden states are defined by the probability to observe an outcome Y_t, and account for the dynamics of the observations in terms of the dynamics of the hidden states. The former is based on the assumption that a given observation Y_t in the sequence is generated by an underlying, latent state S_t. The latter is based on the assumption that the hidden states follow a Markov process. That is, the probability of switching from state i at time point t to state j at $t + 1$ only depends on the current state i at time point t. See Figure S1 for a graphical illustration.

The HMM is defined by three sets of parameters: the initial state probabilities $\boldsymbol{\pi}$, the transition probability matrix (TPM) $\boldsymbol{\Gamma}$ with transition probabilities γ_{ij}, and the state-dependent emission distributions $p(y)$. The initial probability $\pi_i = P(S_1 = i)$ denote the probability of each state $S \in (1, 2, ..., M)$ for $t = 1$. Here, we conveniently assume the initial probabilities equal the stationary distribution implied by the transition probability matrix $\boldsymbol{\Gamma}$.

The transition probability matrix $\boldsymbol{\Gamma}$ with transition probabilities

$$\gamma_{ij} = P(S_{t+1} = j | S_t = i) \tag{S1}$$

denote the probability of switching from state $i \in (1, 2, ..., M)$ at time t to state $j \in (1, 2, ..., M)$ at time $t + 1$.

As cognitive, affective and behavioral (CAB) factors are comprised of continuous data, the state-dependent emission distribution

$$P(Y_t = y | S_t = i) \sim N(\mu_i, \sigma_i^2) \tag{S2}$$

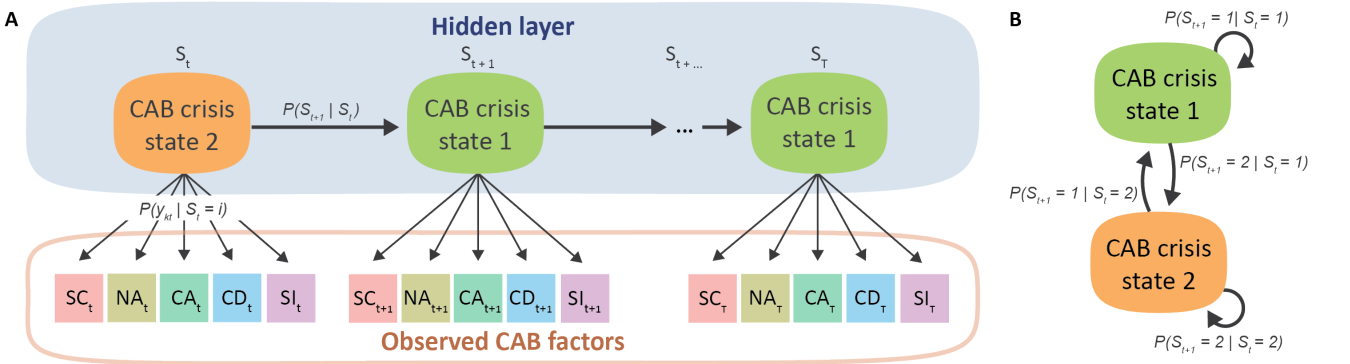

Figure S1: Graphical illustration of the hidden Markov model (HMM; panel A) and temporal dynamics between isolated CAB crisis states (panel B) for an example of a two-state HMM. Here, the hidden (i.e., latent) CAB crisis states $S \in (1, 2)$ over time $t \in (1, 2, \ldots, T)$ are inferred by the observed CAB symptoms self-control (SC), negative affect (NA) contact avoidance (CA) and desire (CD) and suicidal ideation (SI).

denotes the probability of observing y at time t given the hidden state $i \in 1, ..., M$ with state dependent mean μ_i and variance σ_i^2.

To extend the HMM framework to accommodate multivariate data, it is necessary to specify a joint distribution $P(Y_{t1} = y_{t1}, Y_{t2} = y_{t2}, ..., Y_{tk} = y_{tK})$ for the $k \in (1, 2, ..., K)$ state-dependent emission distributions. Here, the shape of the emission distributions can vary over the dependent variables and are typically assumed to be conditionally independent given the sequence of hidden states Zucchini et al. (2017). Thus, the joint distribution can be expressed as the product of the K marginal state-dependent emission probability densities $p_{ki}(y) = P(Y_{tk} = y_{tk}|S_t = i)$:

$$P(Y_{t1} = y_{t1}, Y_{t2} = y_{t2}, ..., Y_{tk} = y_{tk}) = \prod_{k=1}^{K} P(Y_{tk} = y_{tk}|S_t = i) \tag{S3}$$

To allow the inclusion of random effects for the multilevel framework, we follow the implementation in Altman (2007) in which the rows of the transition probability matrix $\mathbf{\Gamma}$ are modeled using a multinomial logit model. Here, the intercepts of the multinomial logit model are composed of a group-level average parameter and an patient specific deviation from that group average, resulting in patient specific transition probabilities. Specifically, the patient n specific transition probabilities γ_{nij} are modeled using:

$$\gamma_{nij} = \frac{\exp(\alpha_{nij})}{1 + \sum_{s=2}^{M} \exp(\alpha_{nis})}, \tag{S4}$$

where

$$\alpha_{nij} = \bar{\alpha}_{ij} + \epsilon_{[\alpha]nij} \tag{S5}$$

for each patient $n \in (1, ..., N)$ and each $i, j \in (1, ..., M)$, with $\bar{\alpha}_{ij}$ being the (group-level) average logit for transitioning from state i to state j, and $\epsilon_{[\alpha]nij}$ denoting the patient n's deviation from that average. The patient level random effects $\epsilon_{[\alpha]nij}$ follow a state i dependent multivariate normal distribution with zero mean vector of length $(M-1)$ and covariance matrix $\mathbf{\Sigma}_{[\alpha]}$ with $(M-1)$ rows and columns.

The patient n, variable k, and state i specific normal emission distributions are composed of a patient n, variable k, and state i specific mean μ_{nki}, and a variable and state dependent variance that is assumed fixed over patients σ_{ki}^2. Similar to the multinomial regression intercepts, the patient n, variable k, and state i specific mean μ_{nki} is composed of a group-level average mean $\bar{\mu}_{ki}$ and an patient specific deviation from that group average $\epsilon_{[\mu]nki}$:

$$\mu_{nki} = \bar{\mu}_{ki} + \epsilon_{[\mu]nki} \tag{S6}$$

The patient level random effects $\epsilon_{[\mu]nki}$ follow a state i and outcome variable k dependent zero mean multivariate normal distribution and variance τ_{ki} .

We adopted a hierarchical Bayesian specification for the parameters in the multilevel HMM (see e.g., de Haan-Rietdijk et al. (2017)). A convenient hyper-prior on the hyper-parameters of the group-level prior distribution is a multivariate Normal distribution for the group-level intercepts $\bar{\alpha}_{i.}$ and an Inverse Wishart distribution for the covariance $\mathbf{\Sigma}_{[\alpha]i.}$. That is,

$$S_{n,t+1} \sim \gamma_{ni.} \text{ with } \gamma_{ni.} \sim MNL(\alpha_{ni.}) \tag{S7}$$

$$\alpha_{ni.} \sim N(\bar{\alpha}_{i.}, \mathbf{\Sigma}_{[\alpha]i.}) \tag{S8}$$

$$\bar{\alpha}_{i.} \sim N(\mathrm{a}_{0i.}, 1K_0\mathbf{\Sigma}_{[\alpha]i.}) \tag{S9}$$

$$\mathbf{\Sigma}_{[\alpha]i.} \sim IW(\mathbf{\Sigma}_{0[\alpha]i.}, df_0) \tag{S10}$$

$$\tag{S11}$$

where the patient-specific probability distribution of the current state $S_{n,t}$ is given by the i row of the transition probability matrix $\mathbf{\Gamma}_{ni.}$ corresponding to the previous state in the hidden state sequence $S_{n,t-1}$. The parameters $\mathrm{a}_{0i.}$ and K_0 denote the values of the parameters of the hyper-prior on the group-level intercepts vector $\bar{\alpha}_{i.}$. Here, $\mathrm{a}_{0i.}$ represents a vector of $(M-1)$ means and K_0 denotes the number of hypothetical patient on which the prior mean vector $\mathrm{a}_{0i.}$ is based. The parameters $\mathbf{\Sigma}_{0[\alpha]i.}$ and df_0, respectively, denote values of the covariance and the degrees of freedom of the hyper-prior Inverse Wishart distribution on the group variance $\mathbf{\Sigma}_{[\alpha]i.}$ of the patient-specific random intercepts $\alpha_{ni.}$.

For the specification of the priors on the continuous emission distributions, we adopt Normal hyper-priors on the group-level means $\bar{\mu}_{ki}$, and Inverse Gamma distributions on the (hyper-) priors on the variances σ^2_{ki} and τ_{ki},

$$y_{nkt} \sim N(\mu_{nki}, \sigma^2_{ki}) \tag{S12}$$

$$\mu_{nki} \sim N(\bar{\mu}_{ki}, \tau_{ki}) \tag{S13}$$

$$\bar{\mu}_{ki} \sim N(\mu_{0ki}, \tau_{0ki}) \tag{S14}$$

$$\sigma^2_{ki} \sim IG(a_{0ki}, b_{0ki}) \tag{S15}$$

$$\tau_{ki} \sim IG(c_{0ki}, d_{0ki}) \tag{S16}$$

The hyper-parameters μ_{0ki} denote the a priori expectations on the group-level mean CAB factors, along with the a priori expectations on their variability, τ_{0ki} over patients. The hyper-parameters a_{0ki} and b_{0ki}, and c_{0ki} and d_{0ki} denote the shape and rate of the Inverse Gamma chosen for the prior on the fixed-over patients' variances σ^2_{ki} of the Normal emission distribution, and the hyper-prior on the between-individual variability τ_{ki}, respectively. These parameters can be used to regulate their degree of informativeness of the (hyper-) priors.

REFERENCES

Zucchini W, Macdonald IL, Langrock R. *Hidden Markov Models for Time Series: An Introduction Using R, Second Edition* (2017). doi:10.1201/b20790.

Altman RM. Mixed hidden markov models: an extension of the hidden markov model to the longitudinal data setting. *Journal of the American Statistical Association* **102** (2007) 201–210. doi:10.1198/016214506000001086.

de Haan-Rietdijk S, Kuppens P, Bergeman CS, Sheeber L, Allen N, Hamaker E. On the use of mixed markov models for intensive longitudinal data. *Multivariate behavioral research* **52** (2017) 747–767. doi:10.1080/00273171.2017.1370364.

2 BLUEPRINT JOINT CRISIS PLAN (JCP) TRANSLATED FROM DUTCH TO ENGLISH

An overview of the complete JCP used as Experience Sampling Method items is given below. ESM items select for the current study are highlighted in grey.

Part 1: At this moment..

Theme	Personalizing options	Answer
1. Sadness (negative affect)	•I feel sad<br>•I feel depressed<br>•I feel dejected<br>•I feel aggrieved<br>•I feel lonely<br>•I feel down<br>•I feel misunderstood<br>•I feel rejected	0 (not at all) – 100 (very much)
2. Happiness (positive)	•I feel happy<br>•I feel merry<br>•I feel positive<br>•I feel cheerful<br>•I am able to enjoy things	0 (not at all) – 100 (very much)
3. Anger (negative affect)	•I feel angry<br>•I feel irritated<br>•I feel frustrated<br>•I feel that others do not take me seriously	0 (not at all) – 100 (very much)
4. Anxiety (negative affect)	•I feel afraid<br>•I feel scared<br>•I feel panicky<br>•I feel hopeless<br>•I am suspicious<br>•I feel that I cannot trust others<br>•I feel left alone	0 (not at all) – 100 (very much)
5. Energy (negative)	•I feel listless<br>•I feel inert<br>•I feel tired	0 (not at all) – 100 (very much)
6. Tense (negative affect)	•I feel tense<br>•I feel restless<br>•I feel irritable<br>•I feel agitated<br>•I feel stressed<br>•I experience chaos<br>•I feel hasty<br>•I feel impatient	0 (not at all) – 100 (very much)

Theme	Personalizing options	Answer
7. Desire to undertake (positive)	•I like to undertake something<br>•I need to do something	0 (not at all) – 100 (very much)
8. Desire for contact (positive)	•I desire contact with others<br>•I would like to be with someone<br>•I want to meet up with someone	0 (not at all) – 100 (very much)
9. Self-image (negative)	•I am useless<br>•I am worthless<br>•I hate myself<br>•I am dissatisfied with myself<br>•I am a failure<br>•I do not deserve to live<br>•I think I am a burden to others<br>•I am insecure	0 (not at all) – 100 (very much)
10 Self-control	•I can handle things<br>•I experience control<br>•I can set boundaries<br>•I can handle life<br>•My life is going well<br>•I oversee my life	0 (not at all) – 100 (very much)
11. Having company (Context)	I am ...	Multiple answers possible:<br>•Alone<br>•With family<br>•With partner<br>•With friends<br>•With people I do not know<br>•With pets<br>•With roommates<br>•With colleagues/classmates<br>•With care providers<br>•With care acquaintances
12. Location (Context)	Where are you?	Multiple answers possible:<br>•At home<br>•At school/work<br>•At the house of family/friends<br>•At a café/library<br>•At a shop/supermarket<br>•Outside in nature (e.g., park/forest)<br>•With care providers<br>•On the go<br>•Other<br>→ *blank space for remarks (obligatory)*

Theme	Personalizing options	Answer
13. Activity (Context)	What are you doing	Multiple answers possible:<br>•Resting/doing nothing<br>•Something peaceful (like reading or watching tv)<br>•Working/studying<br>•Exercising/walking/cycling<br>•Hobby (like making music, doing jobs around the house or drawing)<br>•A trip (like going to the city or to a concert)<br>•Talking to someone<br>•WhatsApp<br>•Social Media<br>•Eating/cooking<br>•Playing games<br>•Something intimate (like cuddling or sex)<br>•Doing chores, groceries or administration<br>•Self-care<br>•Sleeping<br>•Being on the go<br>•Unhelpful activities<br>•Other<br>→ *blank space for remarks (obligatory)*

Part 2: Since the last measurement..

Theme	Personalizing options	Answer
1. Contact avoidance (negative)	•I avoided contact with others<br>•I have isolated myself<br>•I withdrew myself	0 (not at all) – 100 (very much)<br><br>*Including drop down menu (multiple responses possible):*<br>•No WhatsApp<br>•No phone calls<br>•Not answering the doorbell<br>•No texting<br>•Cancelling an appointment<br>•Not going to work/study<br>•Does not apply (only possible if answered with a 0 'not at all')
2. Self-care (positive)	•I took care of myself<br>•I have paid attention to taking care of myself	0 (not at all) – 100 (very much)<br><br>*Including drop down menu (multiple responses possible):*<br>•Showering<br>•Proper day-night rhythm<br>•Healthy meals (3x a day)<br>•Exercising<br>•Medication by prescription<br>•Medication prescribed as a standby taken<br>•Does not apply (only possible if answered with a 0 'not at all')
3. (Self)-destructive	•I have hurt myself or others<br>•I displayed destructive behavior<br>•I have done things that have negative effects on me or others	0 (not at all) – 100 (very much)<br><br>*Including drop down menu (multiple responses possible):*<br>•Cutting myself<br>•Head banging<br>•Scratching myself open<br>•Scraping myself<br>•Fist smashing<br>•Other physical damage inflicted<br>•Smashing stuff<br>•Hair pulling<br>•Substance use<br>•Spending too much money<br>•Gambling<br>•Binge-eating<br>•Gambling<br>•Does not apply (only possible if answered with a 0 'not at all')

Theme	Personalizing options	Answer
4. Suicidal ideation (negative)	•I have considered death	0 (not at all) – 100 (very much)<br><br>*Including drop down menu (multiple responses possible):*<br>•Passively thought about death (for example 'I'd better be dead')<br>•Active suicidal thoughts (for example 'dying is the only option')<br>•Wrote a farewell letter<br>•Specific plans<br>•Does not apply (only possible if answered with a 0 'not at all')
5. Undertaking activities (positive)	•I have undertaken activities<br>•I have done activities<br>•I have been busy	0 (not at all) – 100 (very much)<br><br>*Including drop down menu (multiple responses possible):*<br>•Movie/Netflix/watching tv<br>•Listening to/making music<br>•Yoga<br>•Walking<br>•Doing something creative<br>•Reading<br>•Exercising<br>•Doing chores/ groceries/ administration<br>•Playing games<br>•Sleeping<br>•Having contact on WhatsApp/the phone<br>•Meeting up with someone<br>•Going outside<br>•Other<br>•Does not apply (only possible if answered with a 0 'not at all')

Theme	Personalizing options	Answer
6. Neurotic behavior (negative)	•I have been restless	0 (not at all) – 100 (very much)<br><br>*Including drop down menu (multiple responses possible):*<br>•Nail biting<br>•Pacing<br>•Asking for attention<br>•Cleaning extensively<br>•Smoking extensively<br>•Talking louder or faster<br>•Interrupting others<br>•Worrying<br>•Doing compulsions<br>•Extensive contacting others<br>•Not being alone<br>•Not making decisions<br>•Does not apply (only possible if answered with a 0 'not at all')
7. Psychotic experiences(negative)	•I have had special or unusual thoughts or experiences	0 (not at all) – 100 (very much)<br><br>*Including drop down menu (multiple responses possible):*<br>•Hearing voices<br>•Seeing shadows<br>•Having dissociations<br>•Having suspicious thoughts<br>•Receiving assignments<br>•Experiencing relivings<br>•Does not apply (only possible if answered with a 0 'not at all')
8. Obligations (positive)	•I have fulfilled my daily occupations<br>•I have fulfilled my daily obligations	0 (not at all) – 100 (very much)<br><br>*Including drop down menu (multiple responses possible):*<br>•Work<br>•Study<br>•Taking care of the children<br>•Washing<br>•Cooking<br>•Cleaning<br>•Groceries<br>•Live up to my agreements<br>•Does not apply (only possible if answered with a 0 'not at all')

Theme	Personalizing options	Answer
9. Had/sought contact with others (negative)	•I have been unkind to others<br>•I have argued with others	0 (not at all) – 100 (very much)<br><br>*Including drop down menu (multiple responses possible):*<br>•Telephone<br>•Face-to-face<br>•WhatsApp<br>•Social Media<br>•Does not apply (only possible if answered with a 0 ‘not at all’)
10. Somatics (context)	•I have experienced somatic complains	0 (not at all) – 100 (very much)<br><br>*Including drop down menu (multiple responses possible):*<br>•Breathing difficulties<br>•Pain or tightness of the chest<br>•Palpitations<br>•Nausea<br>•Changes in defecation (constipation/diarrhea)<br>•Palpitations<br>•Nausea<br>•Changes in defecation (constipation/diarrhea)<br>•Stomachache<br>•Headache<br>•Dizziness<br>•Tiredness<br>•Backpain<br>•Muscle-/joint pain<br>•Tingling’s<br>•Sweating<br>•Extensive sleeping<br>•Insufficient sleeping<br>•Nightmares<br>•Does not apply (only possible if answered with a 0 ‘not at all’)
11. Pleasant experiences (positive)	•I have experienced something pleasant	0 (not at all) – 100 (very much)<br>→ *blank space for remarks (obligatory)*
12. Unpleasant experiences (negative)	•I have experienced something unpleasant	0 (not at all) – 100 (very much)<br>→ *blank space for remarks (obligatory)*

Part 3: Current level of tension..

Theme	Personalizing options	Answer
1. Level of tension	Which phase are you currently in…	•Green<br>•Yellow<br>•Orange<br>•Red

3 SUPPLEMENTARY TABLES AND FIGURES

3.1 Tables

Table S1. Demographic and clinical characteristics of the sample.

Demographic or clinical variable	n (%)
Age, $M(SD)$	35.12 (12.18)
Educational attainment	
Completed high school	8 (30.8)
Completed vocational training	8 (30.8)
Completed higher professional	5 (19.2)
Completed university	1 (3.8)
Not listed	4 (15.4)
Comorbidity with non-PD classifications	
Depressive disorder	8 (30.8)
Post-traumatic stress disorder	5 (19.2)
Anxiety disorder	3 (11.5)
Autism spectrum disorder	3 (11.5)
Attention-deficit hyperactivity disorder	2 (7.7)
Substance-use disorder	3 (11.5)
Obsessive-compulsive disorder	1 (3.8)
Anorexia nervosa	1 (3.8)

Note. We did not assess ethnic identification, culture background or a measure of income.

Table S2. Model fit and convergence indices.

	2-state model	3-state model	4-state model	5-state model
AIC	2664.32	2630.36	2616.45	2623.73
Median Rhat (% $>$ 1.2)				
transition parameters	1.02 (0.00%)	1.01 (0.00%)	1.02 (6.25%)	1.04 (16.00%)
composition parameters	1.01 (0.00%)	1.01 (0.00%)	1.03 (0.00%)	1.04 (4.00%)

Table S3. CAB crisis state compositions for the 2-, 3-, 4-, and 5-state model.

		Self-control		Negative affect		Contact avoidance		Contact desire		Suicidal ideation	
	State	Mean	SD	Mean	SD	Mean	SD	Mean	SD	Mean	SD
2-state model	1	33.70	15.90	47.74	14.10	31.04	18.99	37.41	19.39	38.05	13.64
	2	27.90	15.62	61.20	17.34	40.07	28.91	30.09	24.61	54.74	23.01
3-state model	1	34.81	14.59	47.32	12.85	25.98	17.56	41.11	19.46	37.18	11.51
	2	35.06	18.15	56.22	16.23	37.22	25.87	41.35	25.60	51.27	23.16
	3	13.70	8.27	71.98	16.65	47.42	34.07	7.00	3.74	65.34	18.27
4-state model	1	43.54	14.70	31.80	13.23	17.30	16.07	41.38	17.61	3.85	2.40
	2	37.16	14.82	49.84	13.12	28.23	18.68	41.59	21.75	46.51	16.72
	3	30.25	15.37	62.15	13.80	40.75	25.46	40.22	25.16	58.07	19.58
	4	13.30	8.70	72.31	16.17	48.17	33.61	6.67	3.38	65.71	17.17
5-state model	1	45.70	13.95	32.46	12.22	20.54	16.29	42.47	16.86	4.14	2.52
	2	36.54	15.08	46.63	12.95	9.24	4.37	40.81	19.15	38.66	14.67
	3	41.08	17.23	48.78	14.19	37.98	22.28	41.84	24.74	44.98	19.31
	4	26.87	10.99	66.69	11.57	47.03	24.21	38.75	23.98	63.80	17.00
	5	11.64	7.77	73.47	16.46	51.29	34.25	6.13	3.17	66.83	17.32

4 FIGURES

4.1 Model fit checks

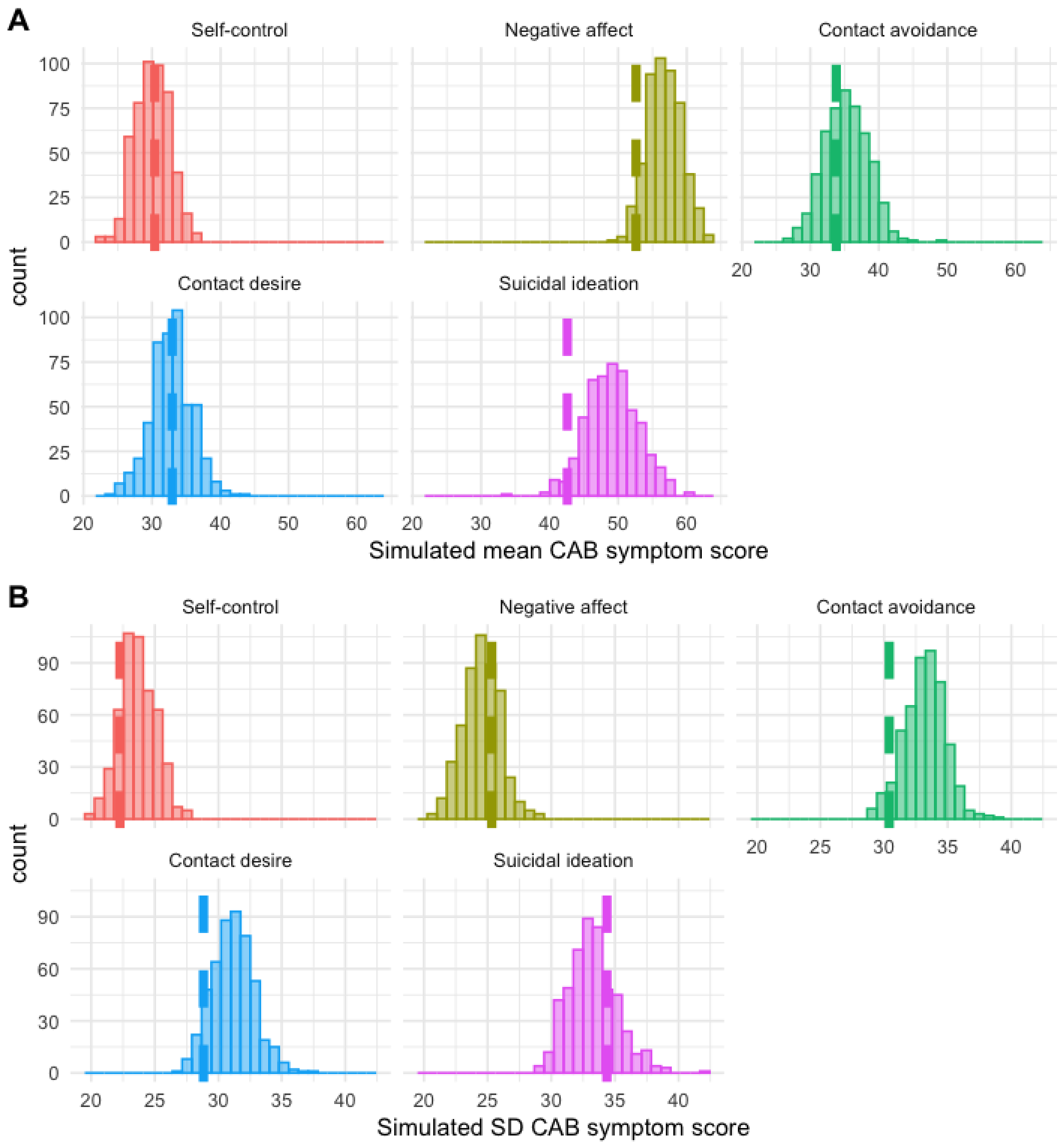

Figure S2: Posterior predictive checks on the group level means (Panel A) and standard deviation (SD; Panel B) of the CAB symptoms self-control, negative affect, contact avoidance, contact desire and suicidal ideation. Each histogram represents the distribution of the variable means (Panel A) or standard deviation (Panel B) over 500 simulated datasets, with superimposed dashed vertical line indicating the actual mean variable score in the empirical ESM data. The synthetic data simulated by the multilevel HMM was able to recover the aggregated mean and standard deviation variable scores of the empirical set, indicating good model fit.

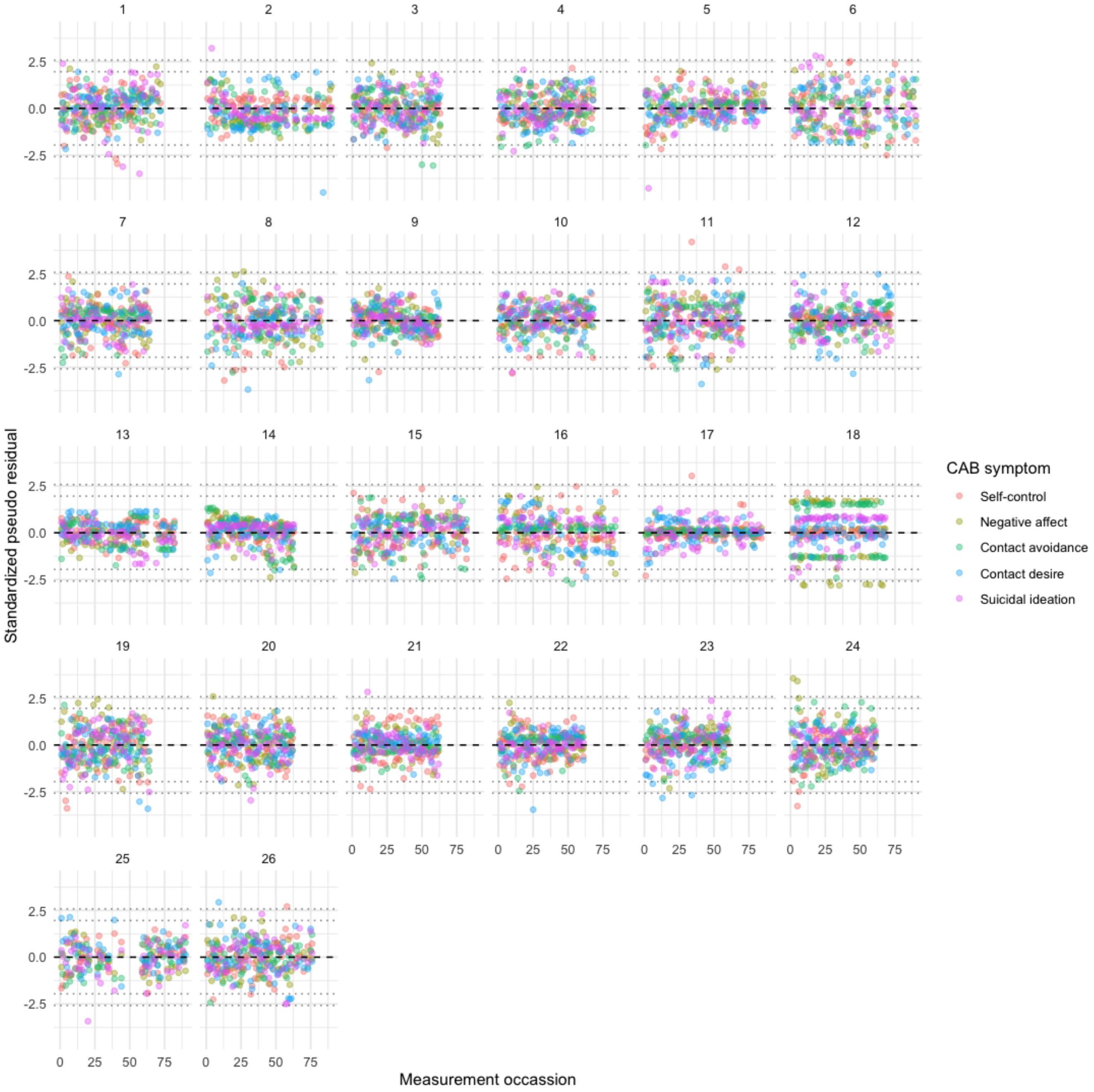

Figure S3: Standardized pseudo residuals over measurement occassions of the CAB symptoms self-control, negative affect, contact avoidance, contact desire and suicidal ideation. Standardized pseudo residuals are displayed for each patient separately. Dashed horizontal line represents the expected zero mean over residuals, dotted line represents 1.96 and 2.58 standard deviations above and below the expected mean. The standardized residuals do not show any trend over time, are approximately divided equally above and below the zero mean, and show few values outside of the 1.96 and 2.58 thresholds, indicating good model fit.

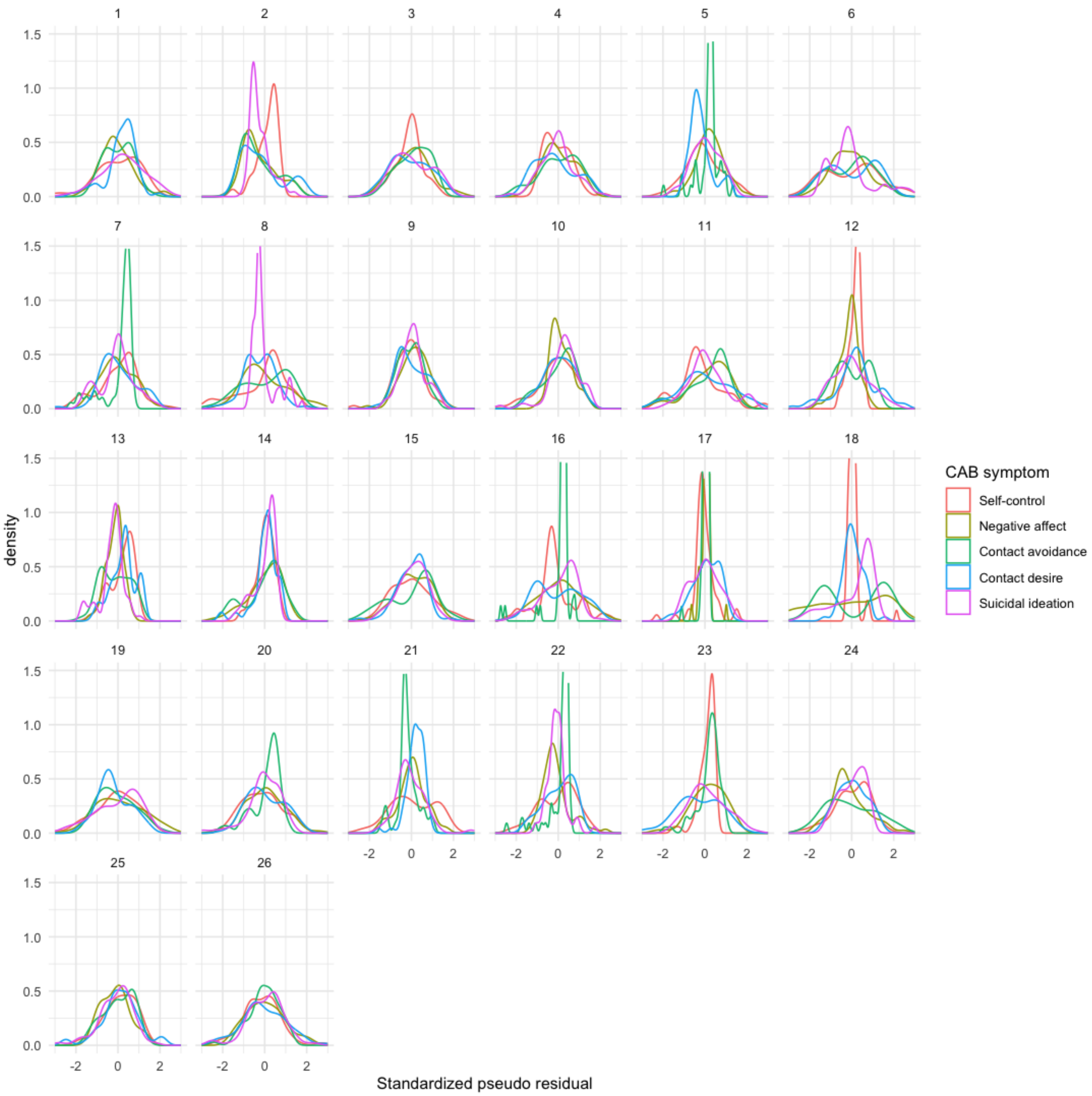

Figure S4: Density plots of the standardized pseudo residuals of the CAB symptoms self-control, negative affect, contact avoidance, contact desire and suicidal ideation. Density plots are displayed for each patient separately. In general, the density plots approximate a normal distribution, indicating good model fit.

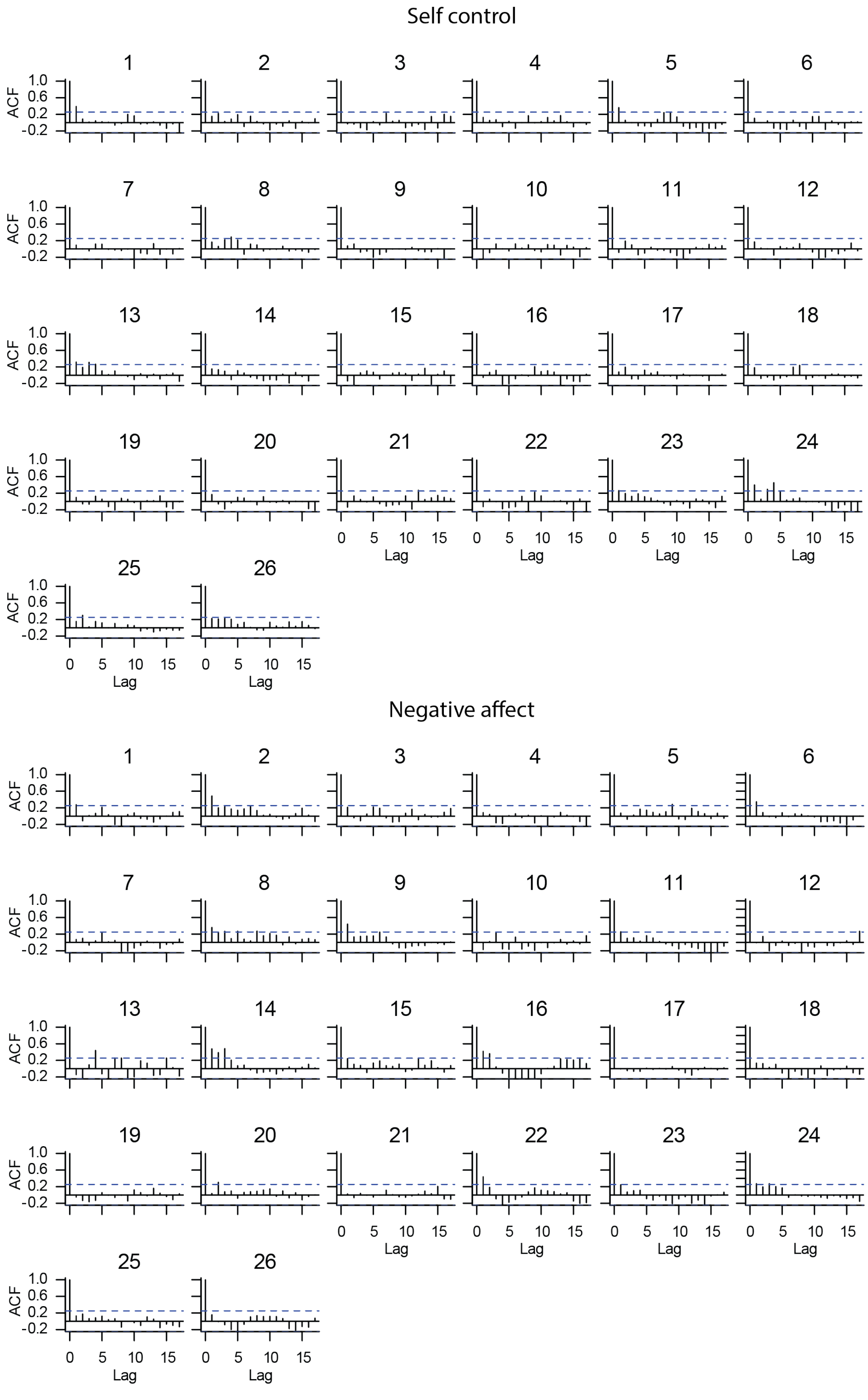

Figure S5: Auto correlation function (ACF) of the standardized residuals of the CAB symptoms self-control and negative affect. The ACF is displayed for each patient separately. The ACF shows values below 0.20 and above -0.20 from a lag of 1 onwards, indicating good model fit.

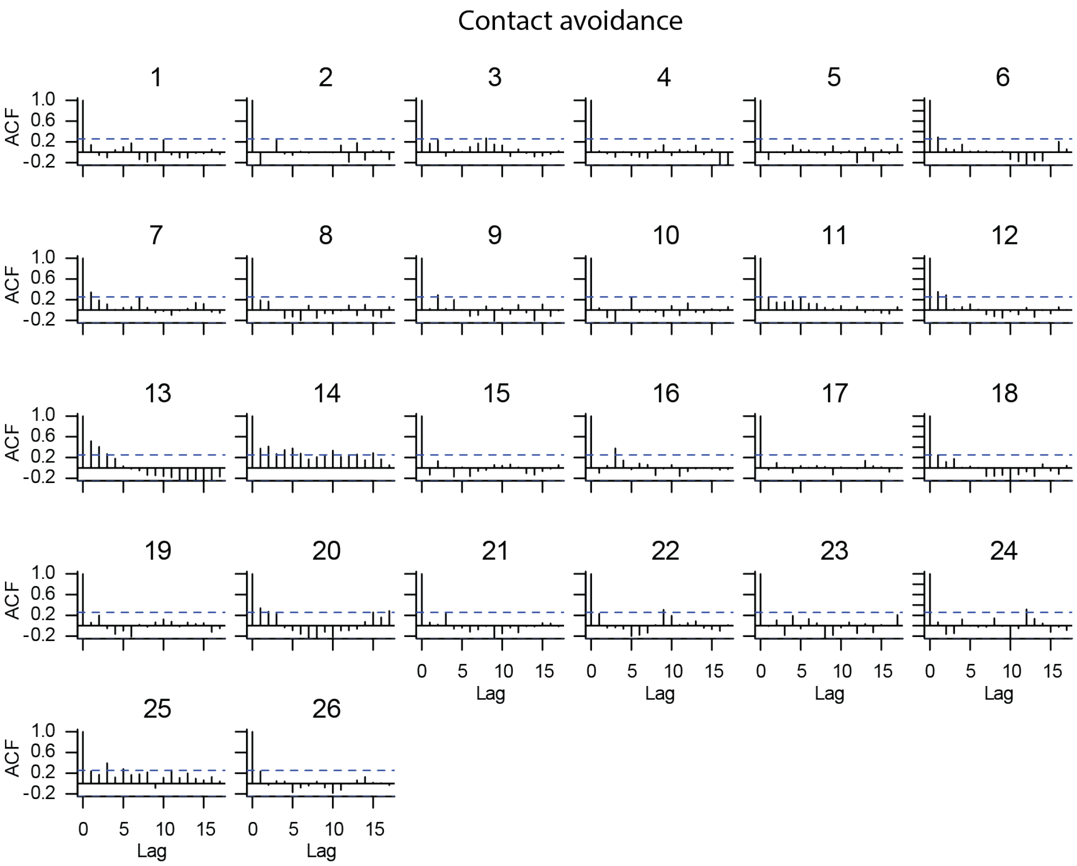

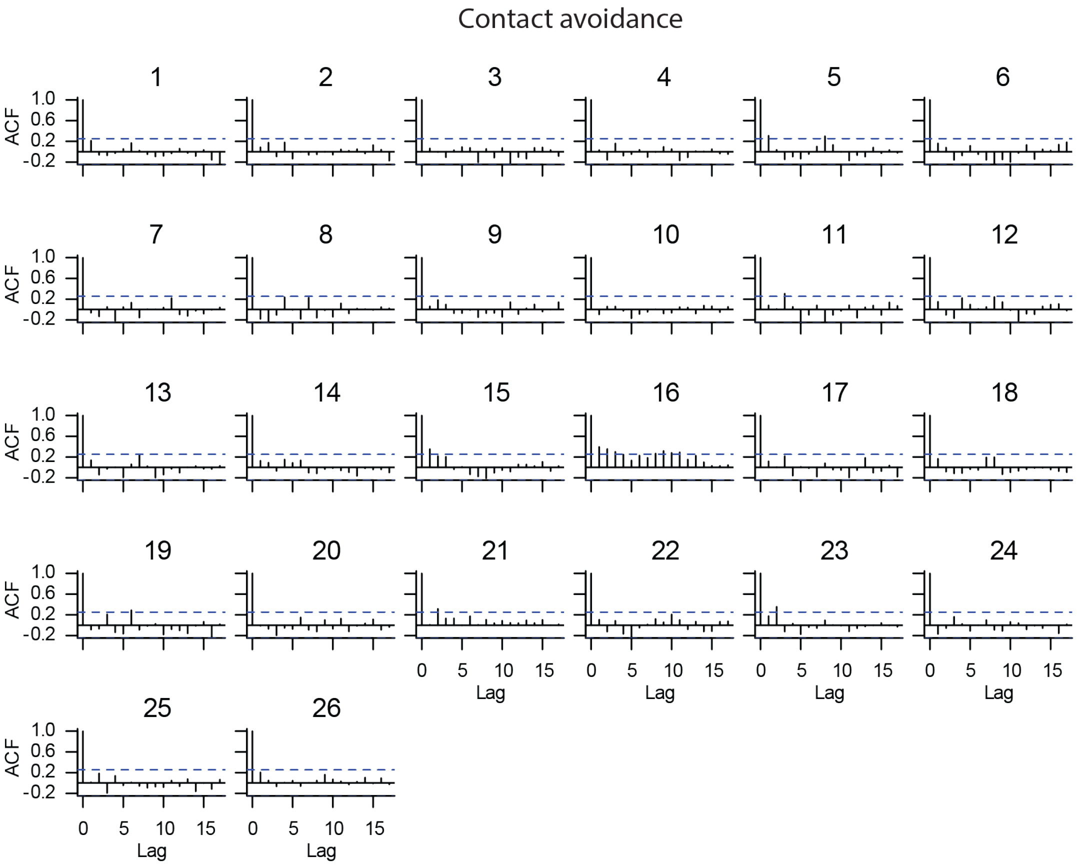

Figure S6: Auto correlation function (ACF) of the standardized residuals of the CAB symptoms contact avoidance and contact desire. The ACF is displayed for each patient separately. The ACF shows values below 0.20 and above -0.20 from a lag of 1 onwards, indicating good model fit.

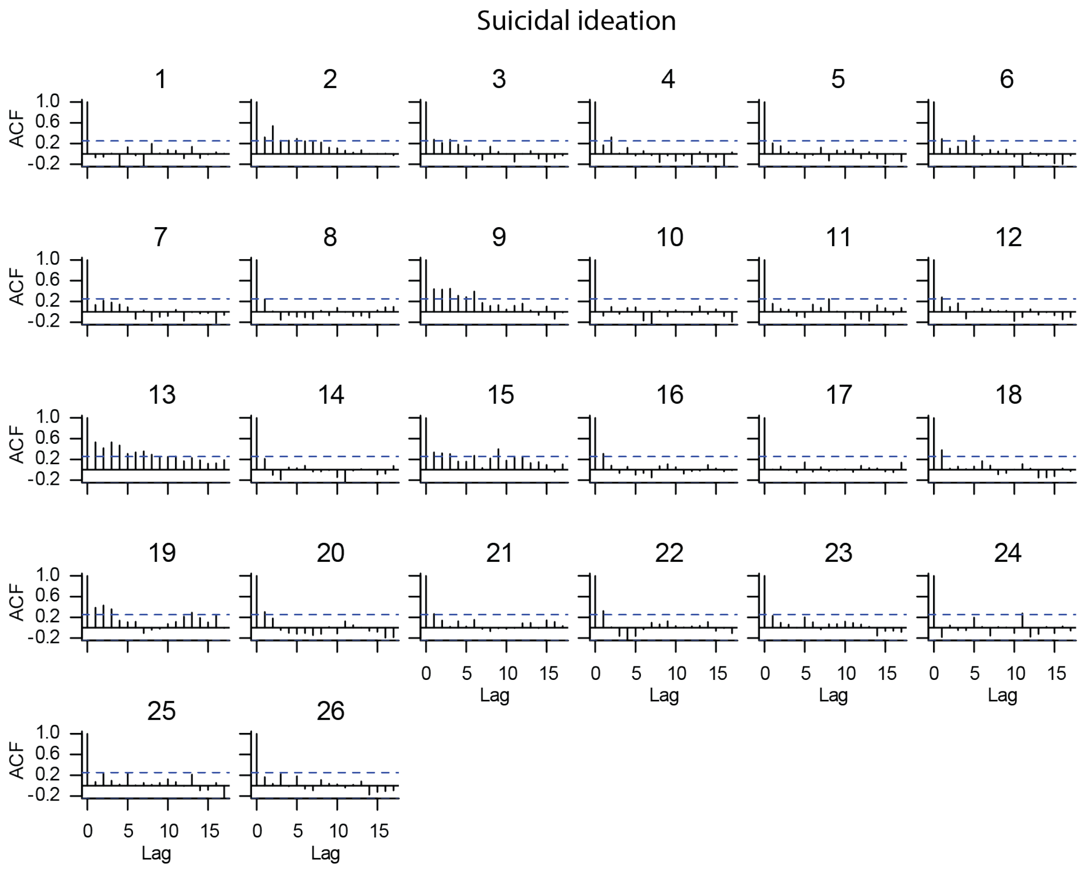

Figure S7: Auto correlation function (ACF) of the standardized residuals of the CAB symptom suicidal ideation. The ACF is displayed for each patient separately. The ACF shows values below 0.20 and above -0.20 from a lag of 1 onwards, indicating good model fit.

4.2 Patient individual model parameters

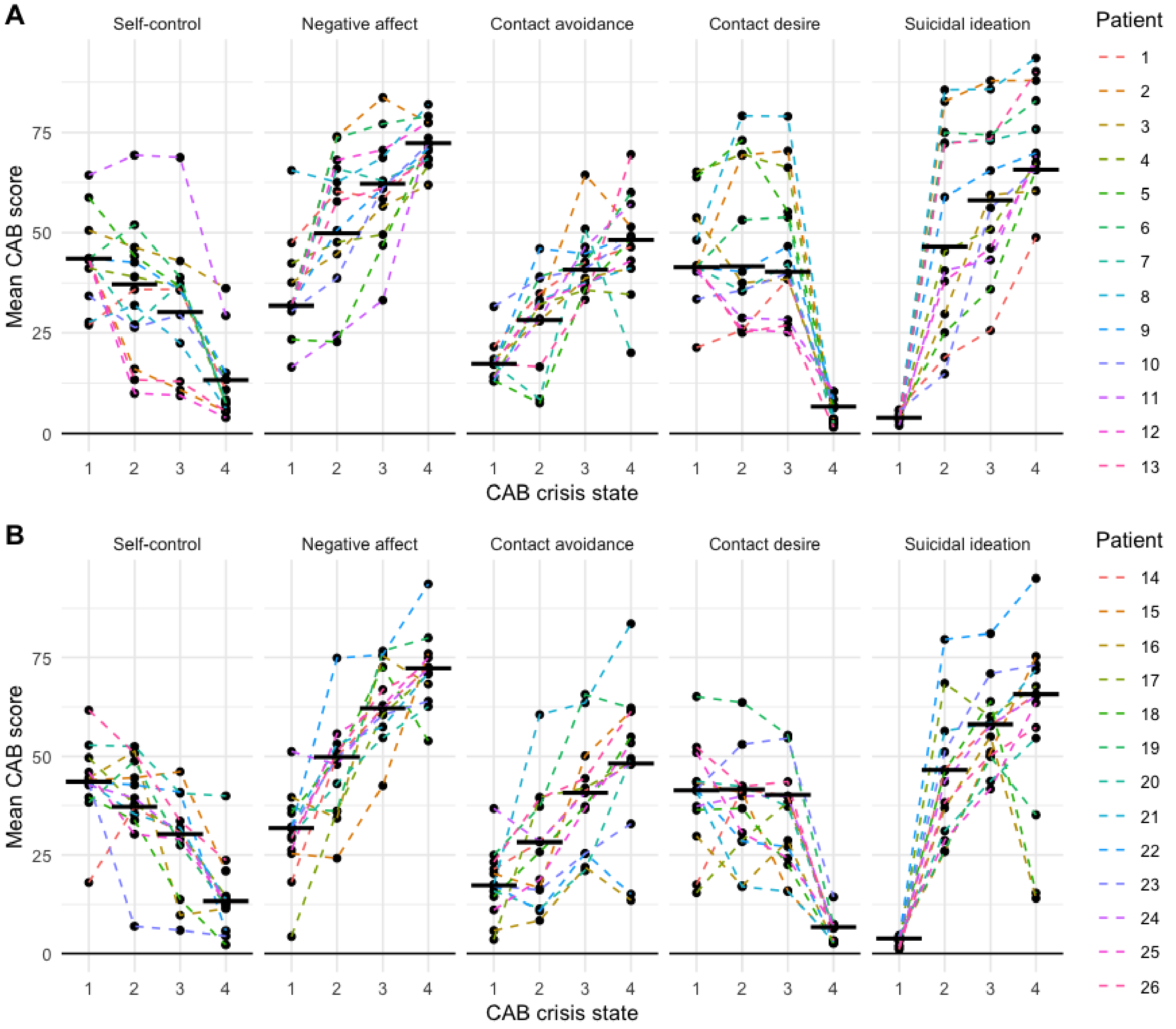

Figure S8: CAB crisis state composition by self-control, negative affect, contact avoidance, contact desire and suicidal ideation with superimposed patient specific CAB crisis state composition pattern (dashed lines). Crisis state dependent means are displayed at the sample- level (horizontal segments) and patient individual level (dots).

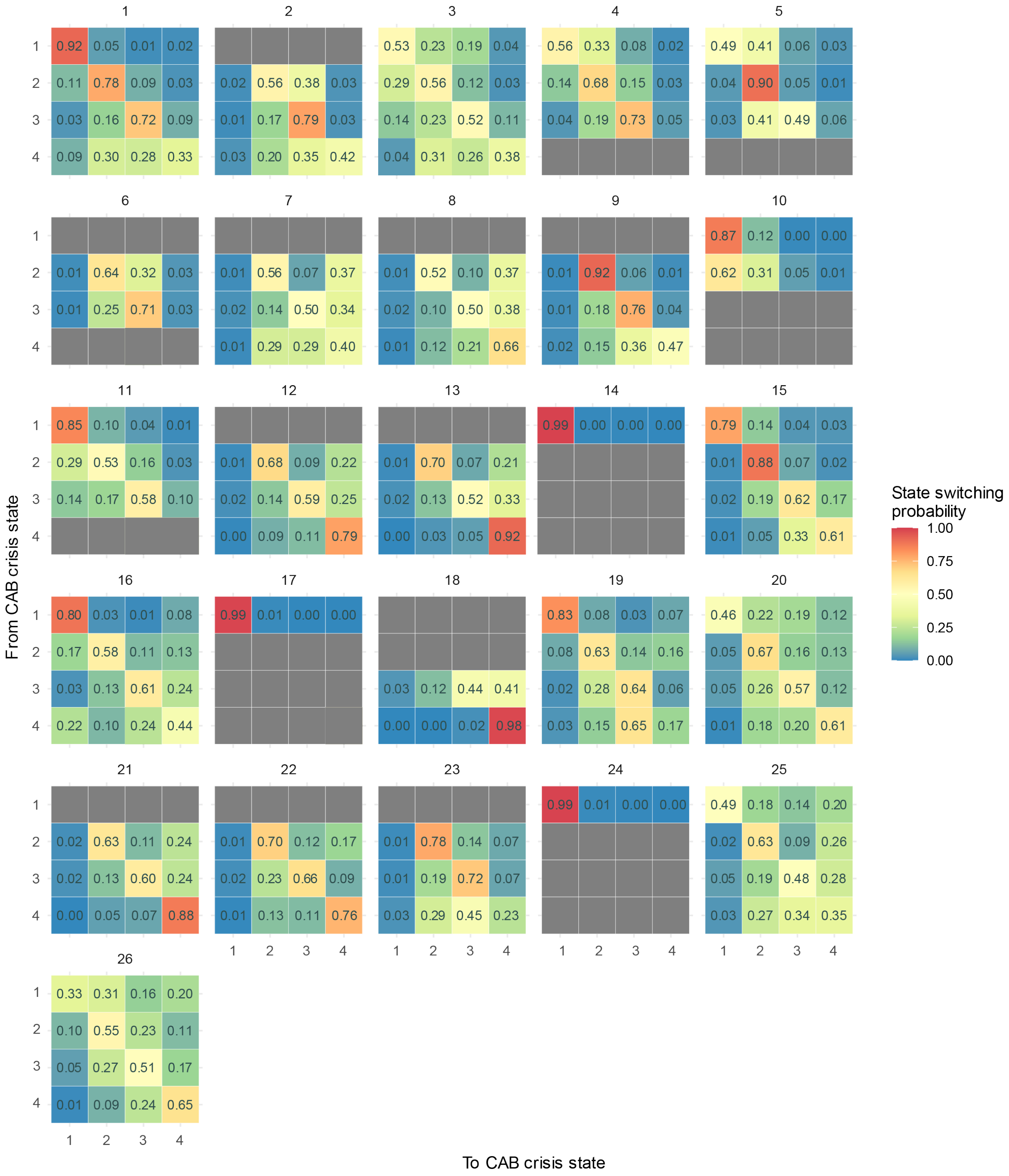

Figure S9: Probabilities of remaining withing or transitioning between the CAB crisis states at the patient individual-level. Probabilities for departing states which, according to the Viterbi algorithm, are not visited or only visited once in the inferred state sequence are omitted at the patient-individual level to convey a representative reflection of the patient-level CAB state dynamics.
